# Imputed State-Level Prevalence of Achieving Goals To Prevent Complications of Diabetes in Adults with Self-Reported Diabetes — United States, 2017–2018

**DOI:** 10.15585/mmwr.mm6945a1

**Published:** 2020-11-13

**Authors:** Yu Chen, Deborah Rolka, Hui Xie, Sharon Saydah

**Affiliations:** 1 Division of Diabetes Translation, National Center for Chronic Disease Prevention and Health Promotion, CDC.

Diabetes increases the risk for developing cardiovascular, neurologic, kidney, eye, and other complications. Diabetes and related complications also pose a huge economic cost to society: in 2017, the estimated total economic cost of diagnosed diabetes was $327 billion in the United States (*1*). Diabetes complications can be prevented or delayed through the management of blood glucose (measured by hemoglobin A1C), blood pressure (BP), and non–high-density lipoprotein cholesterol (non-HDL–C) levels, and by avoiding smoking; these are collectively known as the ABCS goals (hemoglobin A1C, Blood pressure, Cholesterol, Smoking) (*2*–*5*). Assessments of achieving ABCS goals among adults with diabetes are available at the national level (*4*,*6*); however, studies that assess state-level prevalence of meeting ABCS goals have been lacking. This report provides imputed state-level proportions of adults with self-reported diabetes meeting ABCS goals in each of the 50 U.S. states and the District of Columbia (DC). State-level estimates were created by raking and multiple imputation methods (*7*,*8*) using data from the 2009–2018 National Health and Nutrition Examination Survey (NHANES), 2017–2018 American Community Survey (ACS), and 2017–2018 Behavioral Risk Factor Surveillance System (BRFSS). Among U.S. adults with diabetes, an estimated 26.4% met combined ABCS goals, and 75.4%, 70.4%, 55.8%, and 86.0% met A1C <8%, BP <140/90 mmHg, non-HDL–C <130 mg/dL and nonsmoking goals, respectively. Public health departments could use these data in their planning efforts to achieve ABCS goal levels and reduce diabetes-related complications at the state level.

This analysis included adults aged ≥20 years who reported having received a diagnosis of diabetes (excluding gestational diabetes) from a health care provider. This report defined ABCS goals as A1C <8%,* BP<140/90 mmHg, non-HDL–C <130 mg/dL, and being a nonsmoker (*4*). Nonsmokers were defined as those who provided negative responses to questions about smoking (≥100 cigarettes in their lifetime and being a current smoker at the time of the survey). To estimate state-level prevalence, the raking method^†^ was first used to adjust BRFSS weights to the ACS on age, sex, race, health insurance status, education, and income to reflect the state population characteristics (*7*). Multiple imputation methods were used (*8*) to predict the values of A1C, BP, and non-HDL–C for adults with self-reported diabetes in the weight-adjusted BRFSS data.^§^ Variables common to both NHANES and BRFSS were used as predictors (i.e., age, sex, race, health insurance status, education, income, body mass index category, and health status).^¶^ Prevalence was estimated by averaging the estimates from all imputed data sets,** and standard errors were pooled by combining the within-imputation variance and the between-imputation variance (*8*). For the nonsmoking goal, the state-level prevalence was estimated directly from weight-adjusted BRFSS data. The national prevalence of each of the ABCS goals was a direct estimate from 2015–2018 NHANES. For prevalence of achieving ABCS goals, 90% confidence intervals (CIs) were calculated to help illuminate meaningful differences while reflecting the uncertainty inherent in these modeled estimates. Analyses were conducted using SAS software (version 9.4; SAS Institute). This activity was reviewed by CDC and was conducted consistent with applicable federal law and CDC policy.^††^

Among adults with self-reported diabetes, 26.4% met combined ABCS goals nationally, and state-level estimates ranged from 22.3% to 28.2% (Table). The lowest prevalence was in Wisconsin, and the highest was in Utah. Most of the states varied within the 90% CI of the national prevalence.

**TABLE:** Estimated prevalence* of achieving hemoglobin A1C, blood pressure, cholesterol, and avoiding smoking (ABCS) goals among adults with self-reported diabetes — United States, 2017–2018

Area	Prevalence, % (90% CI)				
	ABCS goals^†^	A1C <8%	BP <140/90 mmHg	Non-HDL–C <130 mg/dL	Nonsmoking
**Nationwide^§^**	**26.4 (22.5–30.3)**	**75.4 (72.7–78.1)**	**70.4 (67.4–73.4)**	**55.8 (51.7–59.9)**	**86.0 (83.6–88.4)**
Alabama	24.5 (21.2–27.7)	75.4 (72.7–78.1)	69.0 (65.9–72.0)	57.9 (53.7–62.1)	85.5 (83.8–87.2)
Alaska	25.4 (18.5–32.4)	77.2 (71.2–83.2)	74.8 (68.3–81.3)	54.4 (47.6–61.1)	87.4 (84.4–90.5)
Arizona	24.2 (21.3–27.2)	74.8 (71.3–78.4)	70.9 (67.7–74.1)	54.7 (51.0–58.4)	87.6 (85.9–89.4)
Arkansas	24.1 (20.8–27.5)	75.1 (71.5–78.8)	70.5 (67.0–74.0)	56.4 (51.2–61.6)	84.1 (81.9–86.3)
California	25.0 (21.8–28.2)	74.6 (71.5–77.8)	71.4 (68.0–74.8)	54.6 (50.2–59.0)	91.0 (89.1–92.8)
Colorado	26.1 (22.5–29.7)	76.8 (73.7–79.9)	72.4 (68.7–76.1)	56.4 (52.9–59.9)	87.9 (86.1–89.6)
Connecticut	24.8 (21.4–28.1)	75.9 (72.8–78.9)	69.6 (66.1–73.1)	56.3 (51.7–60.9)	87.2 (85.4–89.0)
Delaware	24.1 (20.2–28.1)	75.6 (71.6–79.6)	68.4 (64.6–72.2)	56.8 (51.6–61.9)	88.9 (86.9–90.8)
District of Columbia	23.3 (19.6–27.1)	75.1 (70.4–79.8)	62.8 (58.4–67.1)	62.8 (58.0–67.6)	84.4 (81.8–87.0)
Florida	25.0 (21.4–28.6)	75.8 (72.0–79.5)	68.2 (64.7–71.7)	55.6 (51.9–59.3)	89.2 (87.3–91.1)
Georgia	24.5 (21.3–27.7)	74.6 (71.0–78.2)	69.3 (66.2–72.3)	57.5 (54.0–61.0)	87.0 (85.1–89.0)
Hawaii	24.8 (21.2–28.5)	75.1 (71.7–78.4)	69.5 (65.3–73.7)	54.1 (48.8–59.5)	88.4 (86.3–90.5)
Idaho	25.5 (21.1–29.9)	76.3 (72.2–80.3)	72.1 (67.7–76.6)	55.4 (49.5–61.2)	87.7 (85.5–90.0)
Illinois	25.0 (20.9–29.1)	75.3 (71.7–78.8)	70.1 (66.7–73.5)	56.1 (52.0–60.2)	87.9 (85.9–90.0)
Indiana	23.8 (21.6–26.0)	76.3 (73.7–78.9)	70.3 (68.0–72.6)	55.9 (52.4–59.5)	83.6 (82.0–85.2)
Iowa	23.7 (21.0–26.5)	75.8 (72.1–79.5)	69.6 (66.6–72.5)	53.9 (50.9–56.8)	87.2 (85.7–88.7)
Kansas	24.3 (22.0–26.6)	75.6 (73.4–77.7)	71.1 (68.8–73.4)	55.6 (52.1–59.0)	86.2 (84.8–87.5)
Kentucky	24.3 (21.3–27.2)	75.3 (72.1–78.5)	71.2 (68.2–74.3)	57.3 (54.2–60.4)	80.9 (78.8–83.1)
Louisiana	24.5 (20.2–28.8)	75.4 (72.0–78.8)	69.0 (65.4–72.7)	59.2 (53.7–64.6)	85.1 (82.6–87.5)
Maine	25.3 (21.7–29.0)	75.8 (73.1–78.5)	71.3 (68.7–73.9)	56.1 (51.3–60.8)	85.8 (83.9–87.7)
Maryland	26.5 (23.8–29.3)	76.4 (73.2–79.7)	70.1 (67.3–72.9)	58.2 (55.1–61.3)	88.5 (86.8–90.2)
Massachusetts	26.2 (21.9–30.4)	76.3 (72.3–80.2)	71.3 (67.3–75.3)	56.3 (50.3–62.2)	87.5 (85.0–90.0)
Michigan	24.6 (22.2–27.1)	76.1 (72.5–79.8)	69.6 (66.6–72.6)	56.2 (53.2–59.3)	86.3 (84.8–87.8)
Minnesota	25.6 (23.2–28.1)	76.3 (73.9–78.6)	71.2 (68.8–73.7)	56.1 (52.8–59.5)	87.6 (86.3–89.0)
Mississippi	23.5 (20.6–26.4)	74.5 (71.6–77.3)	66.8 (63.3–70.2)	58.7 (55.3–62.1)	84.3 (82.4–86.1)
Missouri	24.7 (20.1–29.3)	75.8 (72.8–78.8)	71.6 (68.1–75.2)	56.7 (52.4–61.0)	83.0 (80.8–85.2)
Montana	25.2 (20.9–29.5)	76.0 (72.2–79.8)	70.6 (65.9–75.4)	54.3 (49.6–59.0)	87.5 (85.4–89.7)
Nebraska	25.5 (22.4–28.6)	76.1 (73.7–78.6)	71.0 (67.9–74.2)	55.1 (51.5–58.7)	89.3 (88.0–90.6)
Nevada	23.2 (17.6–28.8)	75.1 (69.7–80.5)	69.2 (64.4–74.0)	55.9 (48.9–62.9)	85.9 (82.5–89.4)
New Hampshire	26.9 (22.4–31.4)	76.2 (71.1–81.3)	72.0 (68.0–75.9)	55.9 (51.3–60.6)	89.2 (87.3–91.1)
New Jersey	26.2 (21.1–31.3)	76.2 (71.7–80.7)	70.1 (65.4–74.8)	55.3 (50.8–59.9)	88.4 (85.9–91.0)
New Mexico	24.6 (21.1–28.1)	73.9 (69.9–77.8)	71.3 (67.5–75.2)	54.8 (49.7–59.9)	86.4 (84.5–88.4)
New York	23.5 (20.5–26.5)	74.6 (71.1–78.0)	69.6 (66.5–72.8)	55.8 (52.0–59.7)	88.4 (87.0–89.8)
North Carolina	24.6 (20.4–28.7)	76.0 (72.2–79.8)	69.2 (65.4–73.1)	57.5 (53.0–62.1)	84.1 (81.1–87.1)
North Dakota	23.7 (21.1–26.3)	75.9 (72.1–79.7)	71.6 (68.4–74.8)	55.1 (51.2–58.9)	82.4 (79.7–85.0)
Ohio	24.6 (22.1–27.1)	76.0 (73.8–78.1)	69.9 (67.4–72.4)	55.8 (52.7–59.0)	84.7 (83.1–86.3)
Oklahoma	24.8 (21.8–27.8)	75.7 (72.9–78.5)	72.0 (68.7–75.4)	54.6 (50.7–58.5)	85.4 (83.6–87.3)
Oregon	24.8 (21.3–28.3)	76.1 (71.8–80.4)	72.6 (68.1–77.1)	54.9 (50.7–59.2)	84.2 (81.3–87.0)
Pennsylvania	25.3 (21.7–28.9)	76.0 (72.2–79.7)	70.3 (66.5–74.1)	56.6 (51.4–61.9)	85.4 (83.3–87.5)
Rhode Island	25.2 (21.1–29.2)	75.9 (72.1–79.7)	70.5 (66.8–74.1)	55.3 (50.4–60.3)	87.7 (85.5–89.8)
South Carolina	24.6 (22.3–26.9)	74.9 (72.3–77.4)	67.9 (65.7–70.1)	58.4 (55.9–60.9)	85.2 (83.5–86.9)
South Dakota	24.5 (20.1–28.8)	76.2 (71.4–81.0)	70.0 (65.2–74.8)	55.3 (49.0–61.6)	84.0 (80.3–87.7)
Tennessee	22.5 (18.6–26.4)	74.9 (71.5–78.4)	69.8 (66.1–73.5)	56.7 (52.8–60.5)	79.3 (76.7–81.9)
Texas	23.5 (19.4–27.6)	73.7 (69.3–78.0)	70.1 (63.9–76.3)	54.9 (49.8–60.0)	88.4 (85.6–91.1)
Utah	28.2 (25.1–31.3)	76.0 (72.3–79.6)	73.6 (70.1–77.1)	54.7 (50.6–58.8)	91.8 (90.3–93.4)
Vermont	25.4 (21.8–29.1)	75.6 (71.8–79.3)	71.2 (67.3–75.2)	57.1 (52.7–61.6)	86.0 (83.4–88.7)
Virginia	25.5 (22.2–28.8)	75.7 (72.1–79.3)	69.3 (65.9–72.7)	56.6 (53.3–60.0)	87.3 (85.7–88.8)
Washington	26.1 (23.8–28.3)	76.2 (73.8–78.6)	72.8 (70.2–75.4)	54.7 (51.9–57.5)	89.4 (88.1–90.8)
West Virginia	24.0 (20.6–27.5)	75.1 (72.2–78.1)	73.5 (70.5–76.5)	56.2 (52.5–59.9)	80.7 (78.8–82.6)
Wisconsin	22.3 (18.2–26.3)	76.0 (72.2–79.8)	70.1 (65.7–74.4)	52.8 (47.4–58.2)	85.5 (83.0–88.1)
Wyoming	24.0 (19.9–28.1)	75.0 (69.6–80.4)	72.6 (69.3–76.0)	53.2 (48.9–57.6)	86.1 (83.7–88.5)

**Abbreviations:** A1C = hemoglobin A1C; ACS = American Community Survey; BP = blood pressure; BRFSS = Behavioral Risk Factor Surveillance System; CI = confidence interval; NHANES = National Health and Nutrition Examination Survey; non-HDL–C = non–high-density lipoprotein cholesterol.

* State-level estimates of A1C, BP, and non-HDL–C were created by raking and multiple imputation methods using data from 2009–2018 NHANES, 2017–2018 ACS, and 2017–2018 BRFSS; state-level estimates of nonsmoking were estimated directly from weight-adjusted BRFSS data, and the raking method was first used to adjust 2017–2018 BRFSS weights to the 2017–2018 ACS.

^†^ ABCS goals were defined as A1C <8%, BP <140/90 mmHg, non-HDL–C <130 mg/dL, and avoiding smoking.

^§^ National average prevalence of each goal was a direct estimate from 2015–2018 NHANES.

^¶^ Some state-level estimates were below or above the 90% CI of the national prevalence. ABCS goals: Wisconsin; BP goal: Alaska, District of Columbia, Mississippi, Utah, and West Virginia; non-HDL–C goal: District of Columbia; nonsmoking goal: California, Delaware, Florida, Kentucky, Maryland, Missouri, Nebraska, New Hampshire, North Dakota, Tennessee, Utah, Washington, and West Virginia.

For each ABCS goal, nationally, 75.4% met the A1C goal (<8%), 70.4% met the BP goal (<140/90 mmHg), 55.8% met the non-HDL–C goal (<130 mg/dL), and 86.0% met the nonsmoking goal. Among adults with diabetes who attained the A1C goal, the lowest prevalence was 73.7% (Texas), and the highest prevalence (77.2%) was in Alaska; all were within the 90% CI of the national estimate. The lowest prevalence of meeting the BP goal was 62.8% in DC, and the highest (74.8%) was in Alaska. The lowest prevalence of achieving the non-HDL–C goal was 52.8% in Wisconsin, and the highest was 62.8% in DC. The prevalence in DC was above the 90% CI of national prevalence. The lowest prevalence of achieving the nonsmoking goal (79.3%) was in Tennessee, and the highest (91.8%) was in Utah. When comparing the individual goals (Figure), the prevalence of achieving the nonsmoking goal was the highest, and that of achieving the non-HDL–C goal was the lowest. In addition, there was a relatively larger variation among states in achieving the nonsmoking goal than other goals.

**FIGURE:**
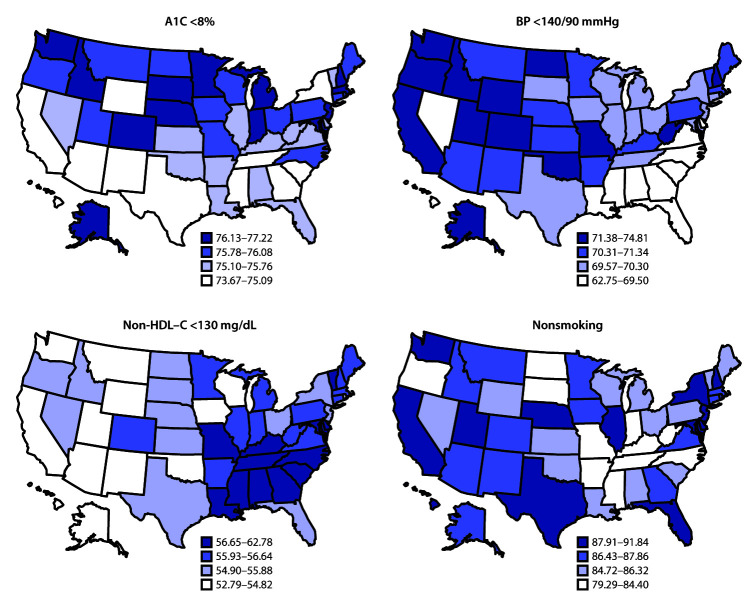
Estimated prevalence* of achieving individual goals of ABCS^†^ among adults with self-reported diabetes — United States, 2017–2018 **Abbreviations:** A1C = hemoglobin A1C; ABCS = hemoglobin A1C, blood pressure, cholesterol, and avoiding smoking; BP= blood pressure; non-HDL–C= non-high-density lipoprotein cholesterol. * The percentage intervals for the quantile cutoffs vary because of variations in the distribution of goal achievement. ^†^ ABCS goals were defined as A1C <8%, BP <140/90 mmHg, non-HDL–C <130 mg/dL and avoiding smoking (current smokers were defined as those who had ≥100 cigarettes in their lifetime and were a smoker at the time of the survey).

## Discussion

This is the first study to estimate the state-level prevalence of achieving ABCS goals to prevent complications of diabetes among adults with self-reported diabetes for all 50 U.S. states and DC. The study identified some states where achievements of the ABCS goals are relatively higher or lower.

Previous studies looked at the achievement of ABCS goals among persons with diabetes at the national level. One analysis using the 2007–2012 NHANES data found that among adults with diagnosed diabetes, 21.3% met all ABCS goals, and 63.7% met the goal for A1C, 65.5% for BP <140/80 mmHg, 56.6% for low-density lipoprotein cholesterol <100 mg/dL, and 80.6% for nonsmoking (*6*). The results of the National Diabetes Statistics Report showed that during 2013–2016, 19.2% of adults aged ≥18 years with diagnosed diabetes met goals for A1C <7.0%, BP <140/90 mmHg, non-HDL–C <130 mg/dL, and nonsmoking; 36.4% met goals of A1C <8.0%, BP <140/90 mmHg, non-HDL–C <160 mg/dL, and nonsmoking (*4*).

Achieving goals for ABCS can reduce the risks for diabetes complications. An analysis from the UK Prospective Diabetes Study suggested that among persons with type 2 diabetes, an intensive blood glucose control regimen reduced A1C levels by 11% over 10 years and reduced the risk for microvascular complications by 25% (*3*). In addition, accumulating evidence has shown that reducing BP and cholesterol levels and avoiding smoking help decrease the incidence of cardiovascular complications among persons with diabetes (*5*).

Some potential factors, such as access to health care and the difference in individual sociodemographic factors, might explain the variation in the achievement of ABCS goals. One study found that lack of health care coverage and low use of health care services were associated with poor management of diabetes (*9*). Another study suggested that persons with higher socioeconomic status were more likely to manage diabetes more effectively (*10*).

The findings in this report are subject to at least four limitations. First, the study sample did not include institutionalized adults, who might achieve different levels of reaching ABCS goals than do noninstitutionalized adults. Second, self-reported diabetes status and other variables might be subject to diagnosis, recall, and social desirability bias. Third, the methods applied cannot ensure that all bias was reduced; state-level estimates for A1C, BP, and non-HDL–C levels might be less precise than they would be if these variables had been measured directly, rather than relying on the multiple imputation method. Finally, possible reasons underlying state variation in the prevalence of meeting ABCS goals were not examined.

Despite increased recognition of the importance of effectively managing risk factors among adults with diabetes, the prevalence of meeting ABCS goals to reduce complications of diabetes is still suboptimal. CDC has been working closely with states to address the burden of diabetes. For example, the Diabetes State Burden Toolkit (https://nccd.cdc.gov/Toolkit/DiabetesBurden) provides estimates of the health and economic impact of diabetes by state. In addition, CDC funds state health departments to support programs to help reduce diabetes complications (e.g., Improving the Health of Americans Through Prevention and Management of Diabetes, Heart Disease, and Stroke [DP18–1815]).^§§^ Tracking state-level progress of ABCS levels might help identify gaps in diabetes care. There is a trade-off because direct measurement at the state level is more precise than is imputation but is more costly, whereas imputation is more practical but does not consider variation related to diabetes management programs or policies in states. Nonetheless, public health departments could use these data in their planning efforts to achieve ABCS goal levels and reduce diabetes-related complications at the state level.

**Summary**

**What is already known about this topic?**

Effective management of hemoglobin A1C, blood pressure, cholesterol, and avoiding smoking (ABCS) is important in preventing complications from diabetes. Little information on state-level prevalence in achieving ABCS goals is available.

**What is added by this report?**

During 2017‒2018, the proportion of U.S. adults with self-reported diabetes who met ABCS goals was suboptimal. Only 26.4% met all the ABCS goals, 75.4% met the A1C goal, 70.4% met the blood pressure goal, 55.8% met the cholesterol goal, and 86.0% were current nonsmokers.

**What are the implications for public health practice?**

These estimates provide data that public health departments could use in their planning efforts to achieve ABCS goals and thus reduce diabetes-related complications at the state level.

## Footnotes

* The American Diabetes Association recommends an A1C goal for many nonpregnant adults of <7%, and a less stringent A1C goal of <8% is recommended for persons with other medical conditions and limited life expectancy. https://care.diabetesjournals.org/content/diacare/suppl/2018/12/17/42.Supplement_1.DC1/DC_42_S1_2019_UPDATED.pdf.

† Raking method was used repeatedly by year and by the state to adjust 2017–2018 BRFSS weights to the 2017–2018 ACS. https://journals.sagepub.com/doi/pdf/10.1177/1536867X1401400104.

§ 2009–2018 NHANES was used for conducting multiple imputations.

¶ The study imputed the values of A1C in BRFSS based on the A1C information in NHANES and the shared predictors in NHANES and BRFSS. Similarly, values of BP and non-HDL–C were imputed based on the shared predictors in both data sets and the BP and non-HDL–C information respectively in NHANES.

** The multiple imputation method generated multiple data sets. Each imputed data set was then analyzed individually, and the final result was obtained by combining the results obtained from all the imputed data sets.

†† 45 C.F.R. part 46, 21 C.F.R. part 56; 42 U.S.C. Sect. 241(d); 5 U.S.C. Sect. 552a; 44 U.S.C. Sect. 3501 et seq.

§§ https://www.cdc.gov/diabetes/programs/stateandlocal/funded-programs/dp18-1815.html.
